# IκBζ: an emerging player in cancer

**DOI:** 10.18632/oncotarget.11624

**Published:** 2016-08-26

**Authors:** Marie Willems, Nadège Dubois, Lucia Musumeci, Vincent Bours, Pierre A. Robe

**Affiliations:** ^1^ Department of Human Genetics and GIGA Research center, University of Liège, Liege, Belgium; ^2^ Department of Neurology and Neurosurgery, T&P Bohnenn Laboratory for Neuro-Oncology, Brain Center Rudolf Magnus, University Medical Center of Utrecht, Heidelberglaan, Utrecht, The Netherlands

**Keywords:** IκBζ, nuclear IκB protein, NF-κB pathway, cancer, perspectives

## Abstract

IκBζ, an atypical member of the nuclear IκB family of proteins, is expressed at low levels in most resting cells, but is induced upon stimulation of Toll-like/IL-1 receptors through an IRAK1/IRAK4/NFκB-dependent pathway. Like its homolog Bcl3, IκBζ can regulate the transcription of a set of inflamatory genes through its association with the p50 or p52 subunits of NF-κB. Long studied as a key component of the immune response, IκBζ emerges as an important regulator of inflammation, cell proliferation and survival. As a result, growing evidence support the role of this transcription factor in the pathogenesis number of human hematological and solid malignancies.

## INTRODUCTION

### The NF-κB family of proteins

NF-κB (*Nuclear Factor kappa B*) is a ubiquitous family of transcription factors involved in biological processes such as inflammation, immunity, proliferation and apoptosis [1–3]. This family of proteins comprises two subfamilies that share a DNA-binding and dimerization domain called the Rel homology domain (RHD) [4] and form homo- or hetero- dimers. The first subfamily of proteins (c-Rel, RelB, p65/RelA) contains a C-terminal transactivation domain. The second subfamily of proteins (p105 and p100) has a C-terminal region that contains multiple copies of ankyrin repeats, instead of a transactivation domain, and can bind to and inhibit Rel proteins. p100 and p105 can however undergo limited proteolysis to generate p52 and p50, respectively, which can form heterodimers with Rel proteins to form transcriptional activators [5].

The involvement of NF-κB in the development, the progression and the therapeutic resistance of many human cancers is well established. Constitutive p50/p65 activity is observed in a large variety of hematological as well as solid tumors [6–8], as a result of an aberrant expression of p50/p65, deletions of the IκBα inhibitor gene or an increased IKK activity [9–13]. Through this constitutive activity, NF-κB p50/p65 acts in tumors mainly as an inhibitor of apoptosis [8, 14]. In addition, anti-cancerous agents, such as TNFα, ionizing radiation and chemotherapeutic drugs activate p50/p65 [15, 16] leading to cell survival and consequently to drug resistance.

Several clinical trials using inhibitors of NF-κB activation have been performed, and have shown variable results in a few types of cancers [17–21]. To date, the most significant clinical results have been obtained with bortezomib, an inhibitor of the proteasome, for the treatment of multiple myeloma [22].

### The IκB family of proteins

**Figure 1:**
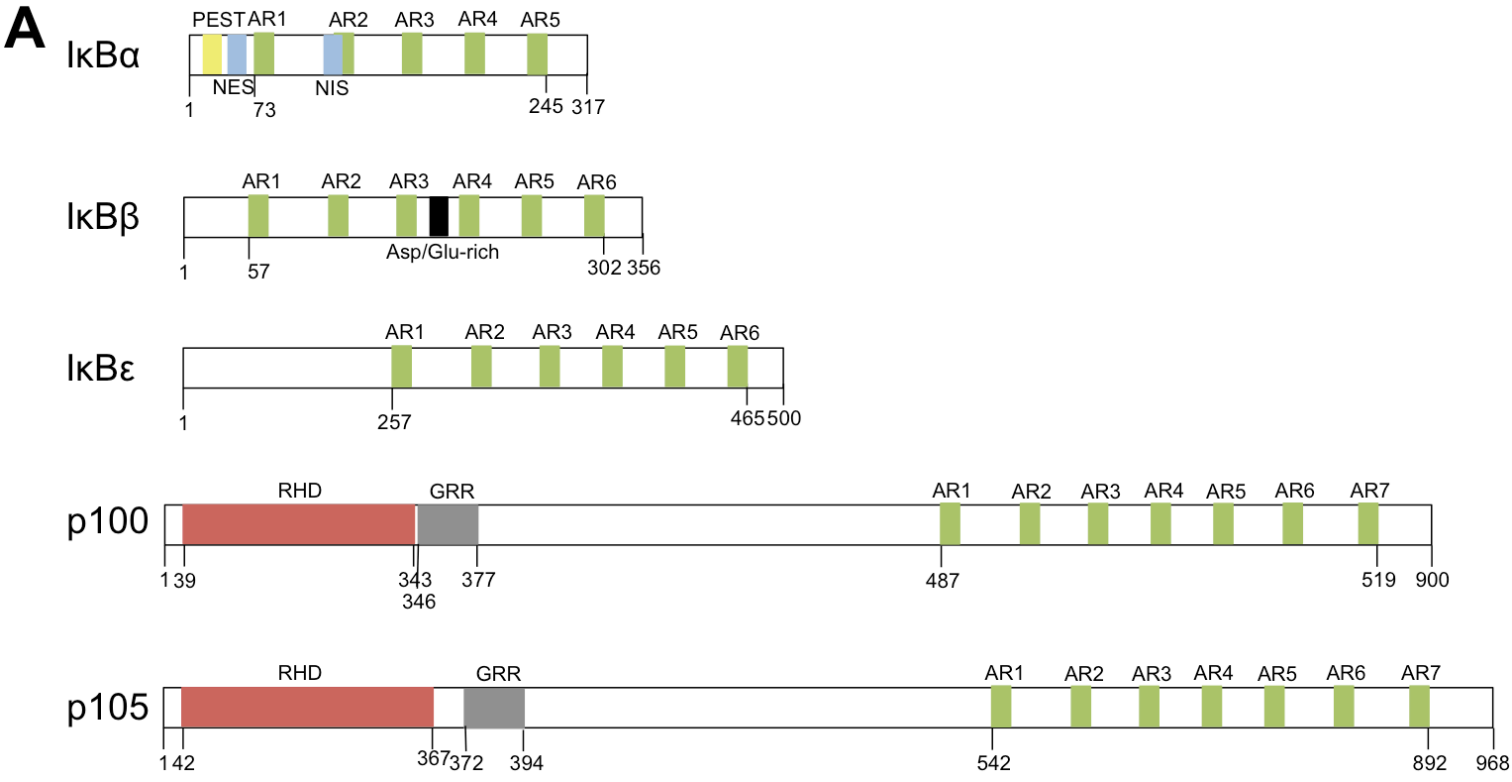

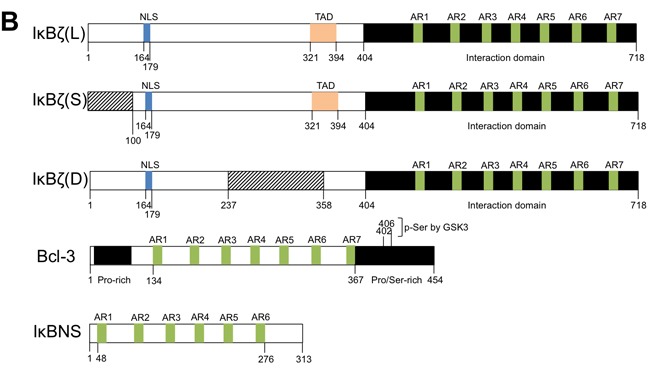
Schematic representation of the IκB family of proteins **A.** The cytoplasmic IκB proteins. Notes: PEST: domain rich in proline, glutamic acid, serine and threonine; AR: ankyrin-repeat; NES: nuclear export signal; NIS: nuclear import signal; RHD: Rel homology domain; GRR: glycine-rich region. **b.** The nuclear IκB proteins. Notes: AR: ankyrin-repeat; NLS: nuclear localization signal; TAD: transactivating domain.

NF-κB protein dimers are kept in the cytoplasm by interaction with proteins of the IκB family (IκB -α, -β and -ε), or by their p100 or p105 component that masks their nuclear localization sequences (NLS, Figure 1, panel A). Upon phosphorylation of specific serine residues, these ankyrin-repeat proteins undergo proteasome- or calpain-dependent complete or limited degradation, allowing the nuclear translocation of the NF-κB protein dimers [23]. The activation of NF-κB occurs *via* either the classical, the alternative, the atypical or the p105-dependent pathways according to the stimuli and the kinases implicated. IκBα, -β and -ε can be phosphorylated by IKKβ (classical pathway), inducing their proteasome degradation. Following UV-irradiation, CK2 can also phosphorylate IκBα, leading to its calpain-dependent degradation (atypical pathway). p100 and p105 phosphorylations respectively depend upon IKKα and IKKβ, themselves activated by NIK. These alternative pathways lead to the activation of RelB/p52 and RelB/p50 pathways, respectively [24, 25].

The IκB family of proteins also comprises additional members (Figure 1, panel B) named nuclear IκB proteins due to the presence of a conserved nuclear localization signal. Unlike the cytoplasmic IκB proteins, the nuclear IκB proteins also harbor a trancriptional activity. Bcl3, which is predominantly expressed in the nucleus, acts as a nuclear transcriptional co-activator or co-repressor that can activate or repress a set of NF-κB target genes through the formation of heterocomplexes with p50 or p52 dimers [26]. Another nuclear IκB protein, called IκBNS, was also shown to be a nuclear transcription factor. IκBNS is a short-lived protein induced by NF-κB activation and its degradation depends upon the proteasome and is regulated by ubiquitin-independent post-traductional modifications of its PEST-domain [27].

## IκBζ

IκBζ, a third member of the nuclear IκB family that shares a strong functional and structural homology with Bcl3 and IκBNS, was discovered in 2000 by Kitamura and collaborators as a new ankyrin repeats-containing protein of unknown function that is induced in the mouse brain in response to LPS and that shares homology with IκB protein [28]. Almost at the same time, Haruta identified the same gene in OP9 stromal cells stimulated with interleukin-1 [29].

IκBζ is encoded by NFKBIZ, *Nuclear Factor Of Kappa Light Polypeptide Gene Enhancer In B-Cells Inhibitor Zeta*. Southern hybridization showed that NFKBIZ is a single-copy gene and is conserved in human, chimpanzee, Rhesus monkey, dog, cow, mouse, rat, chicken and zebrafish. Using fluorescence *in situ* hybridization analysis, human NFKBIZ gene was mapped to chromosome 3q12.3 [30].

Transcription of NFKBIZ produces fifteen alternative mRNA splice and truncated variants, but only three of these mRNA code for a protein. The long IκBζ(L) mRNA variant contains the sequence from 14 exons while the short IκBζ(S) lacks exon 3 which contains the initiation codon of IκBζ(L), and thus encodes from a downstream initiation site a shorter protein lacking the N-terminal 99 amino acids of IκBζ(L). Further investigations are needed to be able to functionnally distinguish these two variants. The third variant, called IκBζ(D), has a large deletion in the central region and results from an additional splicing in the seventh exon. Present as a minor form in cells [31], IκBζ(D) does not possess the TAD (Transactivating domain) and consequently does not have any transcriptional activity (Figure 1, panel B).

### Regulation of IκBζ protein

The IκBζ protein is barely detectable in most resting cells, with the exception of keratinocytes and several mucosal tissues [32, 33]. Its expression is however readily induced in most tissues upon stimulation of Toll-like receptors (TLR) 2, 4,5, 7 and 9 by their exogenic ligands peptidoglycan, bacterial and mycoplasmal lipopeptides, flagellin, CpG oligonucleotides or LPS [28, 34, 35]. Proinflammatory cyokines, such as IL-1β also strongly induces IκBζ *via* its receptor IL1-R [36, 37].

The TLR -with the exception of TLR-3- and IL1-R share similar cytoplasmic domains called TIR (Toll/IL1Receptors) and bind the adaptor protein MyD88. Upon stimulation, MyD88 recruits the serine-threonine kinases IRAK 1 and 4 to the receptor [38]. Activated IRAK4 then phosphorylates IRAK1, inducing its dissociation from the receptor complex and allowing its interaction with TRAF-6. TRAF-6 in turn activates MAP3K7/TAK-1 which activates the NIK/IKK/IκB/NF-κB as well as the MAPK pathways [39, 40]. The induction of IκBζ is completely abolished in MyD88^−/−^ embryonic fibroblasts [35], by several NF-κB drug inhibitors, or by the overexpression of IκB-α [34]. MAP kinase inhibitors on the contrary do not prevent the induction of IκBζ, indicating that the three MAP kinases, Erk, JNK and p38 kinases are dispensable in this process.

While necessary, the activation of NF-κB is however not sufficient for the activation of IκBζ, and an additional step of mRNA stabilization is required. Indeed, the overexpression of p65 or the activation of NF-κB and MAPK by TNFα barely increase IκBζ protein expression [34, 37] and the short half-life of the IκBζ mRNA (30 min) increases after stimulation with LPS or IL-1β, but not after TNFα receptor activation [41].

**Figure 2:**
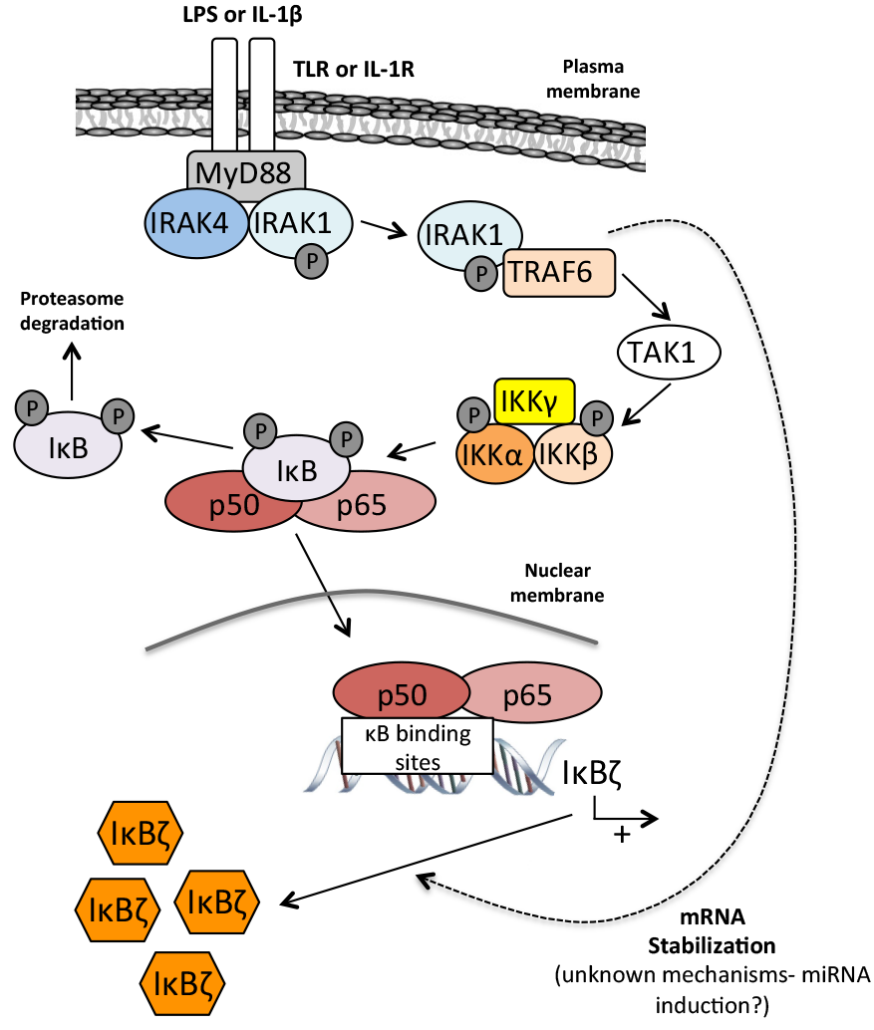
Stable induction of IκBζ Barely detectable in resting cells, IκBζ is induced by lipopolysaccharide (LPS) and IL-1β. Both Toll-like receptor (TLR) and IL-1R share a similar cytoplasmic TIR domain that binds the MyD88 adaptator protein. Under stimulation, MyD88 recruits IRAK1 and IRAK4 leading to the dissociation of IRAK1 and its binding to TRAF6. The complex IRAK1/TRAF6 activates then TAK1 which in turn induces NF-κB translocation. The mRNA stabilization of IκBζ depends upon the recruitment of IRAK1 to the TIR domain of the IL-1R and TLR receptors as well as on a 165-nucleotides sequence present in the 3′-UTR of the IκBζ mRNA. Notes: LPS: lipopolysaccharides; IL-1β: interleukin 1β; TLR: toll-like receptor; IL-1R: IL-1 receptor; IRAK1/4: interleukin-1 receptor-associated kinase 1/4; TRAF6: TNF receptor-associated factor 6; TAK1: transforming growth factor beta-activated kinase 1; IκB: inhibitor of κB ; IKK: IκB kinase.

This mRNA stabilization depends on the recruitment of IRAK-1 and TRAF-6 to the TIR domain of IL1-R and TLR receptors [42] (Figure 2) and on a 165-nucleotide cis-element present in the 3′-UTR of the IκBζ mRNA (*Untranslated region*) [43]. This cis-element contains four AU-rich elements (AREs) that are the recognition signals for an mRNA processing pathway restricted to certain lymphokines, cytokines and proto-oncogenes [44]. The stabilization of IκBζ mRNA does however not respond to the same stimuli as that of cytokines, and the overexpression of HuR [45] or Apobec-1 [46], the transacting factors that bind ARE to stabilize the mRNA of these cytokines, does not affect the stability of the IκBζ mRNA. The exact post-trascriptional regulatory mechanism that leads to IκBζ mRNA stabilization *via* its cis-element remains thus largely unknown, although some recent findings may provide some clues.

Recently for instance, the micro-RNA miR-124a was found to directly target IκBζ mRNA by base pairing to a partially complementary sequence in the 3′UTR, called 7mer (7 nt sites that match the seed region of the miRNA). As a result, miR-124a can suppress IκBζ expression through translational repression [47]. Likewise, *in silico* data suggest that other miRNAs could regulate the stability of IκBζ mRNA as well [48].

Little is known about the post-translational regulation of IκBζ activity. Immunoprecipitation experiments indicate that transfected IκBζ strongly associates with p50/p50 and p50/p65 complexes. IκBζ preferentially binds the p50 subunits of these complexes and its association with the p65 subunit has to date exclusively been detected after overexpression of both proteins [37]. This preferential binding to the p50 subunit is reminiscent of that of Bcl3 [49] and IκBNS [50, 51]. IκBζ, like Bcl3, was also recently shown to associate with p52 in ABC DLBCL (activated B-cell-like subtype of diffuse large B-cell lymphoma) [52]. Like other nuclear IκB proteins, IκBζ regulates the transcriptional activity of NF-κB by forming a stable ternary complex with the subunits of NF-κB and κB sites in the nucleus [53]. The details of the formation of these ternary complexes between IκBζ, NF-κB and the DNA is not yet completely understood. This interaction however appears to be independent from the DNA sequences flanking the NF-κB binding site but involves both the C-terminal extremity of IκBζ, which interacts with the subunits of NF-κB linked to the DNA, and its N-terminal NLS [54, 55]. Of note, experimental IκBζ mutants defective for their NLS localize in the cytosol and inhibit NF-κB like conventional IκB proteins [37, 56]. Whether such a phenomenon also occurs in physiological conditions is to date unknown.

**Figure 3:**
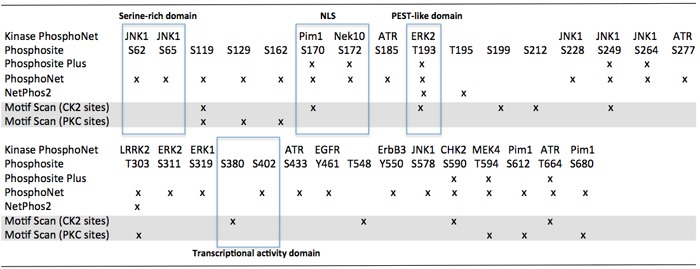
In silico analysis of serine/thréonine and tyrosine- containing motives in IκBζ functional domains Notes: JNK1: c-Jun N-terminal kinase 1; Pim1: serine/threonine-protein kinase pim-1; NeK10: NIMA-related kinase 10; ATR: ataxia telangiectasia and Rad3 related; ERK2: extracellular signal-regulated kinase 2; LRRK2: leucine-rich repeat kinase 2; EGFR: epidermal growth factor receptor; ErbB3: erb-b2 receptor tyrosine kinase 3; CHK2: checkpoint kinase 2; MEK4: mitogen-activated protein kinase kinase 4; PKC: protein kinase C; CKII: casein kinase 2.

It is currently unknown whether IκBζ phosphorylation, ubiquitination or other post-translational protein modifications alter its interactions with NF-κB nuclear or cytoplasmic complexes. *In silico* analyses, however, reveals the presence of several serine/threonine or tyrosine- containing motives for casein kinase 2, EGFR, Chck2, ATR and MAP kinases in functional domains of the protein (Figure 3).

### IκBζ and gene transcription

**Figure 4:**
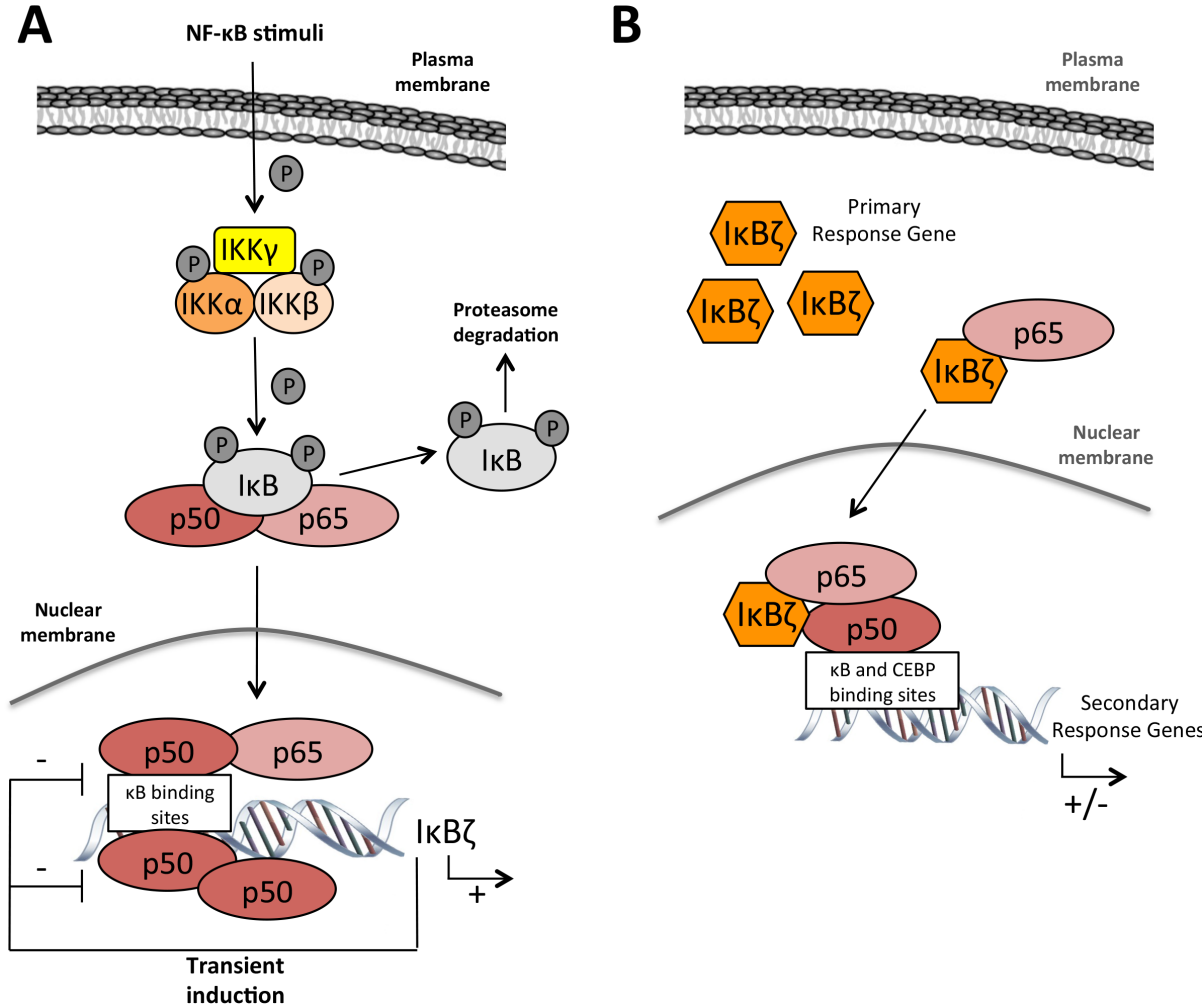
IκBζ function in gene régulation **A.** After NF-κB activation through diverse stimuli, IκBζ is transcribed and transiently induced. Under these conditions, IκBζ acts as a inhibitor of the homodimer p50/p50 or the heterodimer p50/p65 through a negative feedback loop **B.** Upon specific stimulation with LPS or IL-1β leading to IκBζ mRNA stabilization and protein expression, IκBζ forms a ternary complex with p50 and p65 on the promoter of target genes and activates or repress their transcription. Notes: IKK: IκB kinase; IκB: inhibitor of κB; NF-κB: nuclear factor of κB.

Like its homolog Bcl3 that can either induce or repress gene transcription depending on the cellular context and through its association with the p50 or p52 subunit of NF-κB [57], IκBζ can both promote or inhibit gene expression [56, 58] (Figure 4).

Under transient stimulation, IκBζ inhibits the activity of NF-κB by preventing the binding of this transcription factor to the DNA in the nucleus. Detailed electrophoretic mobility shift assays using a probe harboring a canonical NF-κB binding sequence showed that the DNA-binding activity of the NF-κB p65/p50 heterodimer or p50/p50 homodimer was inhibited by the C-terminal ankyrin-repeats of a IκBζ [37]. As such, IκBζ can participate in the control of NF-κB through a negative feedback loop [59]. Likewise, IκBζ can inhibit the DNA binding of, STAT3, another key transcription factor which acts downstream of the JAK-STAT (*Janus kinase/signal transducer and activator of transcription*) pathway to regulate cell proliferation and apoptosis [60].

IκBζ can however also activate the transcription of a set of genes (Table 1, [28, 35, 53, 58, 61–66]). Since IκBζ has no obvious DNA binding motif, and since no consensus structural feature has been found among the promoter sequences of IκBζ-regulated genes, it is unlikely that IκBζ directly associates with DNA to activate gene transcription. It more likely stabilizes or assists the promoter binding of other transcription regulators.

**Table 1:** Confirmed IκBζ target genes

	Regulation	Partners	Cell types	References
IL-6	+	p50; p65	Swiss 3T3 cells; Monocytes	28; 53; 61
hBD2	+	p50	HBE1	62
NGAL	+	NF-κB	A549	63
CCL2	+	NF-κB	Raw264.7	64
IFNγ	+	p50; p65	Lymphocytes; NK cells; HEK 293; KG-1; Monocytes	65; 66
GM-CSF	+	?	Macrophages	35
M-CSF	+	?	Macrophages	35
TNFα	-	p50	HeLa; COS-7; HEK 293	58
IL-12	+	?	Macrophages	35

Notes: IL-6/12: interleukin 6/12; hBD2: human beta-defensin 2; NGAL: neutrophil gelatinase-associated lipocalin; CCL2: chemokine ligand 2; IFNγ: interferon gamma; GM/M-CSF: granulocyte-macrophage/macrophage colony-stimulating factor; TNFα: tumor necrosis factor alpha. Positive (+) or negative (−) transcriptionnal regulation of targeted genes by IκBζ.

Reporter gene and chromatin immunoprecipitation assays have indeed shown that the NF-κB and C/EBP(*CCAAT/enhancer-binding protein*) DNA binding sites are minimal elements essential for the IκBζ mediated transcriptional activation of IκBζ-responsive genes [67]. Yamazaki and collaborators also found that the activation of NF-κB, besides being required for IκBζ induction, is also substantially involved in the transcriptional up-regulation of the IκBζ target genes [68]. Gene knockdown experiment using specific siRNAs indicated that p50, which is known to be constitutively bound to NF-κB-driven promoters, and IκBζ could form a core element for transcriptional activation of target genes while p65 transcriptional activity might be required for the full activation of some of them [69].

A study performed on HEK293 cells using a GAL4 fusion protein technique also suggests that an internal fragment of IκBζ rich in glutamines and prolines (amino acids 329 to 402) possesses an intrinsic transcriptional activity. This transcriptional function would mainly proceed *via* the interaction of IκBζ with the NF-κB p50 subunit. The binding of p50 to IκBζ *via* the ankyrin repeats of IκBζ would in this model prevent the C-terminal region of IκBζ from inhibiting the activity of its own N-terminal region [58].

Finally, IκBζ was identified as a selective regulator of H3K4 trimethylation after nucleosome remodeling. H3K4 trimethylation is an histone-modifying reaction that alters the N-terminal tails and core domains of histones to regulate transcription. This epigenetic mechanism has a well known physiological role in the molecular cascades that regulates transcription of genes involved in primary and secondary inflammatory responses [69] and in cancer [70, 71].

### IκBζ, inflammation and immunity

In line with its transcriptional targets (Table 1), the most important known physiological role of IκBζ was demonstrated in innate immunity against common pathogens, through the modulation of genes of the secondary inflammatory response [35]. Besides its main function in innate immunity, some studies suggested a role for IκBζ in adaptative immunity [36, 72]. As an example, it was showed that IκBζ is induced upon stimulation of B cell antigen receptor (BCR) [73, 74].

As a corollary, IκBζ is involved in diseases related to the response of physical and chemical barriers against infectious agents. NFKBIZ gene-invalidated mice show atopic dermatitis-like lesions [32]. Likewise, IκBζ is involved in the epithelial cell cytokine responses observed in asthma due to house dust mite, where allergens induce monocyte IL-1β production triggering an IκBζ-dependent GM-CSF release from human lung epithelial cells [75]. In patients with ulcerative colitis, the expression of lipocalin-2, an essential marker of activity of the disease, is regulated synergically by IL17-A, IL22 and TNFα in an IκBζ-dependent manner [76, 77]. Likewise, an important role of IκBζ was highlighted in various autoimmune diseases, for example in Sjögren's syndrome-like disease [78], Crohn's disease [79], rhumatoid arthritis [80] as well as in psoriasis. For this last disease, a new susceptibility DNA polymorphism (rs7637230, G→A) was found at a locus adjacent to NFKBIZ [81].

## IκBζ IN CANCER

A strong relation exists between inflammation and cancer, as inflammation plays a critical role in tumor initiation and progression but also influences the response to the treatment [82, 83]. The tumor microenvironment contains innate and adaptative immune cells [84] that interact with cancer cells by direct contact or cytokine and chemokine production. The expression of immune mediators as well as the abundance and activation state of infiltrating immune cells therefore influence tumor growth, anti-tumor immune response, tissue invasion and metastasis, as well as the clinical response to chemotherapy or immunotherapy [85, 86]. The well-known role of IκBζ in cytokine production [62, 64, 65] and its expression in various immune cells suggests a possible role of IκBζ in the tumor microenvironment. In support of this hypothesis, chemically-induced skin carninogenesis was found to associate with both a significant inflammatory response and a major induction of NFKBIZ in mice [87].

Bcl3, which is highly homologous to IκBζ, is also directly involved in lymphoproliferative disorders [88–91] and in solid tumors [92]. Bcl3 was for instance found to promote metastasis in ERBB2-driven mammary tumors [93] and to attenuate the efficacy of Temozolomide in glioma cells [94]. High levels of Bcl3 expression have been observed in various solid tumors where it is involved in the control of cell death and proliferation [95–97]. Likewise, IκBζ is activated and overexpressed in ATL (*Adult T cell Leukemia*) induced by HTLV1 (*Human T cell leukemia virus type I*) *via* the oncoprotein Tax [98], suggesting a role in these lymphoid cancers. NFKBIZ was also recently identified in a molecular signature characteristic of mycosis fungoides, the most common type of primary cutaneous T-cell lymphoma (CTCL) [99]. A recent study showed that activated B-cell-like subtype of diffuse large B-cell lymphoma overexpress IκBζ as compared to control B cells and that its downregulation is selectively toxic to these tumor cells [52] through an activation of the the caspase 3 pathway [78]. More recently, both mutations and amplification of the NFKBIZ gene are associated with the occurrence of primary testicular and primary central nervous system lymphomas [100, 101].

High levels of IκBζ expression have also been observed in solid tumors. For instance, Görranson and colleagues described a role for the interaction of IκBζ with the FUS-DDIT3 fusion oncoprotein in the initiation of myxoïd/round cell liposarcomas (MLS/RCLS) through the transcription of NF-κB dependent genes [102]. The tumor-suppressor miR-124a, and miR-223, which target the NFKBIZ mRNA, were recently shown to be silenced in glioblastomas [47, 48, 103], and we have observed that IκBζ is expressed in these glial tumors where it prevents spontaneous cell death (unpublished data).

**Figure 5:**
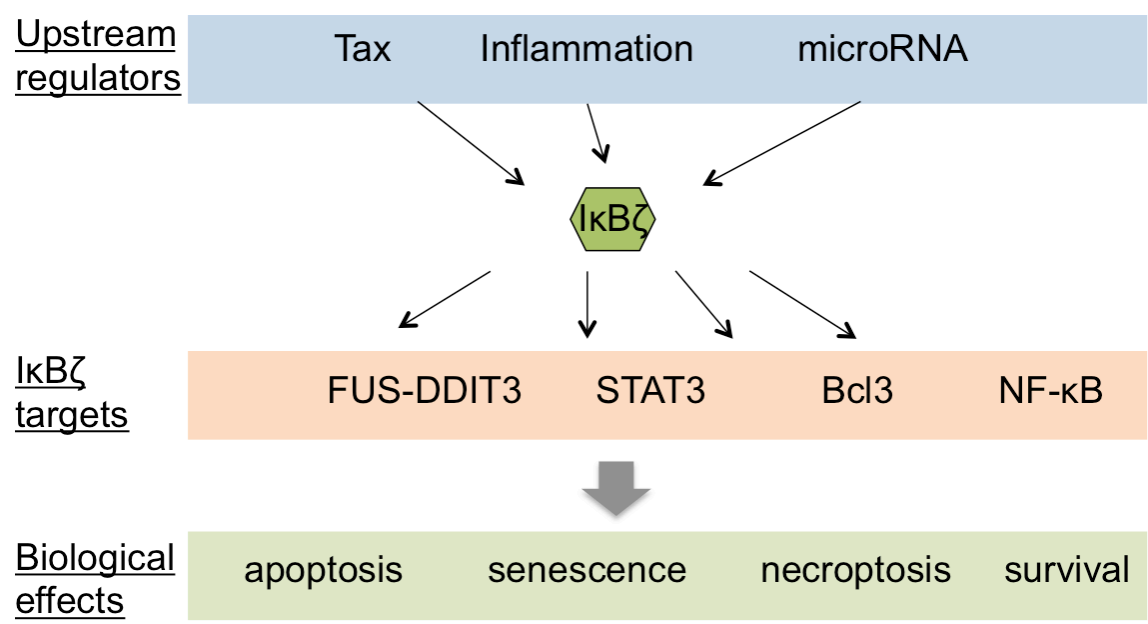
IκBζ and its involvment in cancer Schematic representation of the potential upstream regulators of IκBζ as well as the IκBζ targets and their relative biological effects. Notes: Tax: transactivator of pX; FUS: fused in sarcoma; DDIT3: DNA damage-inducible transcript 3; Bcl3: B cell lymphoma 3; NF-κB: nuclear factor of κB; STAT3: signal transducer and activator of transcription 3.

The biological role of IκBζ in human cancers might however be more intricate than seems at first. This protein indeed modulates altogether cell death, survival and proliferation, and might even work as an oncosupressor in certain tumor types (Figure 5). IκBζ is for instance a regulator of the senescence associated secretory phenotype (SASP) constituted by various growth factors and cytokines secreted by senescent cells, and transgenic IκBζ expression results in enhanced SASP cytokine expression [104]. Wu and collaborators showed that IκBζ physically interacts with and inhibits the transcriptional activity of the oncoprotein Bcl3, leading to apoptosis induction [60]. As mentioned above, IκBζ can also inhibit the activity of the transcription factor STAT3 [60], a transcription factor that is itself frequently overexpressed in tumors, and that regulates the expression of numerous oncogenic genes controlling cell growth and metastasis [105]. Finally, in human fibrosarcoma cells (HT-1080) and breast carcinoma cells (MCF-7/casp-3), the repression of IκBζ with interferent RNA render the cells more resistant to apoptosis, while its overexpression is sufficient to induce cell death [106].

## CONCLUSIONS, THERAPEUTIC POTENTIAL, ONGOING RESEARCH AND UNEXPLORED ASPECTS

IκBζ emerges as an important regulator of inflammation, cell proliferation and survival through its modulation of NF-κB and STAT3 signalings. As such, growing evidence points to the physiopathological role of this transcription factor in a number of hematological and solid malignancies.

Additional tumor specific knowledge is mandatory prior to translating current experimental data to the bedside, given the potentially dual role of IκBζ in cell proliferation and survival. Indeed, IκBζ inhibition can lead either to cell death, in most of cell types, or to cell survival in a few experimental settings. These findings should stimulate further research on the cell-type specific mechanisms regulating IκBζ protein-protein and protein-DNA interactions and pave the way to innovative anti-cancer therapies.
